# Hopes and reality: consumers’ purchase intention towards whitening cream

**DOI:** 10.1186/s43093-021-00098-1

**Published:** 2021-11-01

**Authors:** Md. Monirul Islam, Fathema Farjana Hani

**Affiliations:** 1 Department of Business Administration, Shahjalal University of Science and Technology, Sylhet, 3114 Bangladesh; 2 Department of Business Administration, North East University Bangladesh, Sylhet, 3100 Bangladesh

**Keywords:** Female consumers, Purchase intention, Whitening cream, SEM, Bangladesh

## Abstract

In contemporary Bangladeshi society, popularity, marriage, and status are weighed on a scale where the vital criterion is a fair complexion. Women are encouraged to use whitening cream in its colour-conscious culture. The present study identifies the factors that influence consumers’ purchase intention towards whitening cream. A structured questionnaire was used to elicit feedback from 275 female respondents using the convenience sampling method. Structural equation modelling and hypotheses tests were conducted to validate the model after verifying the scale items’ reliability and validity. The findings revealed that attitude, involvement, and descriptive norms were significant factors, and injunctive norms, perceived quality, and price fairness were inessential factors in explaining Bangladeshi female consumers’ purchase intention. The framework used in the study can assist in product design. The study makes an important contribution to the literature by explaining why female consumers equate whiteness with beauty. Marketers should not use deceptive advertisements to influence them falsely; they should fulfil their expectations without causing harm or inciting racism.

## Introduction

Asians often equate beauty with whiteness, and this concept has been commodified in the marketplace [74]. White skin is pursued as an ideal for many women in Asian cultures [40]. People consider beauty and light skin to be the core of a satisfying life. Females vainly try to reduce melanin by using skin-lightening products [31]. “Skin lightening, also known as skin bleaching and skin whitening, involves the use of topical products that contain corticosteroids, hydroquinone, mercury, and a variety of other agents to attain a lighter skin color”, [71] (p. 349). Despite widespread knowledge that melanin (which produces skin pigmentation) combats the sun’s ultraviolet rays, the use of skin-lightening products has gradually increased.

The idea of skin whitening is intimately associated with personal identity, self-image, and racial identification. Physical attractiveness is regarded as a function of white skin, and the latter has many rewards, for instance, higher self-esteem, better job opportunities and salaries, and physical and mental health [38]. The whitening frenzy abounds amongst females, their families, and society as a whole. This has in part been initiated by marketers. Advertisements show how dusky women can become fairer-skinned if they use whitening cream. Females try to tackle long-standing prejudices and assume more prominence and credibility by using whitening cream. Marketers have been very successful in convincing women of the benefits of having fair skin; whitening cream sales exceed those of Coca-Cola in India.

In Bangladesh, beauty means white skin, and this perception drives the cosmetics industry. According to the Bangladesh Cosmetics and Toiletries Manufacturers’ Association (BCTMA), the cosmetics industry has experienced a compound annual growth rate of 10% in the last 15 years [75]. Despite this, there is a lack of literature on skin-whitening cream in Bangladesh. The present study addresses this shortfall by examining the multiple factors that influence consumers’ purchase intentions in the country. As purchase intention is a crucial factor in assessing consumer behaviour, manufacturers of whitening cream may find the study of assistance when researching consumer perspectives.

## Literature review

### Fairness: the white and the dark side

An Indian non-governmental organisation (NGO) has conducted a campaign called “Dark is Beautiful”. The campaign’s director, Kavitha Emmanuel, said: “Skin colour bias affects people psychologically. It affects how children perform in school because their confidence level goes down; they feel they are not good enough. Moreover, when it comes to marriage, we again find that skin colour plays a vital role. We thought, ‘why are we keeping quiet about this? We should talk about this and see how people respond’”.

Emmanuel delivered a petition containing 30,000 signatures to the Emami cosmetics company protesting their discriminatory advert for their Fair and Handsome brand. However, Emami’s managing director said: “There is a need in our society for whitening creams, so we are meeting the need”. He refused to withdraw it [76].

Colour-sensitive women in Bangladeshi society are so preoccupied with the issue that they apply whitening cream to their children. In Africa, Asia, and Latin American countries, women generally practise skin bleaching because they associate whiteness with social and economic benefits. Educated and working women consider whiteness as a way of competing globally with different races and ethnicities. However, no medical study has proven that whitening cream has permanent results. Not only may whiter skin be out of reach, but the use of such products could also be damaging [77].

### Theoretical framework and hypothesis development

#### Purchase intention

Purchase intention reflects consumer cognitive behaviour regarding the intention to buy specific products or brands [30]. The intensity of customers’ willingness to purchase a commodity is determined by purchase intention. The greater the intensity, the stronger the purchase intention [57]. Moreover, measuring consumer buying behaviour is not straightforward, and purchase intention is crucial to assess consumer buying behaviour. Purchase intention can be used to measure the intensity of attraction towards a particular product. Moreover, stronger purchase intention directs consumers’ desire to buy a product [57]. Consumer willingness and intention to indulge in a transaction is stimulated by purchase intention [50]. Measuring purchase intention is helpful in understanding which brands or products consumers will purchase [23]. Also, it is possible to assess respondents’ attitudes by measuring purchase intention [47].

### Attitudes and purchase intention

Attitude is defined as “a latent disposition or tendency to respond with some degree of favourableness or unfavourableness to a psychological object” [62] (p. 76) and can be applied to purchase intention towards skin-whitening cream. Multi-attribute models such as the theory of reasoned action [3] and the theory of planned behaviour [4] can be used to explore how attitude impacts purchase intention. Attitude towards an object is a consideration in the individual’s evaluation of it. A positive attitude directs them towards an intention to purchase, whereas a negative attitude does the opposite [65]. Attitude is one of the principal determinants of purchase intention. Various researchers have found a connection between attitudes with behavioural intention. For instance, attitude has a positive and significant effect on purchase intention towards halal cosmetic products [1]. It was also revealed how attitudes can predict purchase intention significantly [5]. A person’s attitude towards an object—be it a product or service—can influence their intention to buy or not [3]. A positive attitude towards a product is an indicator of a greater willingness to buy it [16]. Consumers’ intention towards brands is influenced by their attitude, and a positive stance suggests a greater willingness to buy [15]. Positive attitudes are positively associated with consumers’ e-shopping intentions [58]. A study conducted amongst 515 young Chinese consumers found that attitudes had a positive effect on purchase intention towards organic foods [2]. Exploration of consumer attributes amongst Hispanic shoppers of local foods found a positive relationship between attitude and purchase intention [12]. Research also showed how attitude was most significantly related to purchase intention towards local food [59]. In the light of the above, the following hypothesis is proposed:

H1: Consumers with a favourable attitude towards whitening cream have a higher intention to purchase.

### Consumer involvement and purchase intention

Consumer involvement is defined as a person’s perceived relevance of the specific products based upon inherent interest, values, and needs [72]. Product involvement is determined by how far the consumer considers the product to be important to their life [28] and their subjective evaluation of its perceived value [39]. Involvement has been described as the amount of arousal or interest that is generated by a situation, object, or stimulus; it represents an individual-level variable [45]. Product involvement generally refers to consumers’ enduring perceptions of the importance of the product category based on their inherent needs, values, and interests [67]. Customer product selection is determined by purchase intention. The product’s importance to the individual and the latter’s willingness to search for information or alternatives are influenced by their product involvement (Schiffman & Kanuk, 2005). Moreover, higher perceived usefulness directs higher involvement and purchase intention. Consumer involvement is so crucial that it is used to segment consumers [42]. The researchers identified product involvement as an indicator that is linked to product evaluation and purchasing decisions [29]. Because consumer involvement provides motivation to purchase, the consumer is likely to remain longer in a store and visit it more frequently. In other words, it impacts behaviour [66], and this creates positive intention [34]. In the light of the above, the following hypothesis is proposed:

H2: Consumer involvement is positively related to purchase intention towards whitening cream.

### Subjective norms and purchase intention

Subjective norms indicate feelings of morally responsible behaviour in the purchase specific products, and this is dependent on the consumers’ social image [7]. Perceptions of different people’s behaviour (e.g. neighbours, friends, and peers) have a persistent influence on others’ behaviour, and these are considered the subjective norm [4]. Subjective norms reflect perceived social pressure to conform to a specific behaviour and in the present context contribute to forecasting intention to engage in purchasing skin-whitening cream. Previous studies have shown a strong relationship between subjective norms and purchase intention regarding different products; for instance, the intention to engage in e-commerce is influenced by subjective norms. There is a positive relationship between subjective norms and purchase intention of organic products [17]. A positive relationship between subjective norms and consumer purchase intention towards organic foods in particular is also explored [2]. Consumers’ decision-making concerning product choice can be based on the influence of family and friends [70]. Also, consumers’ community involvement and subjective norms positively influenced the intention to use e-commerce websites [37].

A meta-analysis of the theory of planned behaviour exposed that injunctive and descriptive norms are conceptually different constructs [44]. Conceptualisation of injunctive norms alongside the theory of planned behaviour is extensively used [62]. Injunctive norms are defined as the reflection of social pressure regarding one’s conduct through the perception of others’ approval or disapproval [18].

By contrast, descriptive norms are determined by others’ social pressure and what is perceived as normal conduct regarding a behaviour [18]. “A majority of descriptive norms increased self-identification, positive attitudes, and self-efficacy regarding vegetable intake behaviour” [61] (p. 245). These ideas can be adapted to examine intention towards purchasing skin-whitening cream. In the light of the above, the following hypotheses are proposed:

H3: Injunctive norms are positively associated with intention to purchase whitening cream.

H4: Descriptive norms are positively associated with intention to purchase whitening cream.

### Perceived quality and purchase intention

Perceived quality is the estimation made by the consumer relying on the whole set of basic as well as the outer dimension of the product or the service [25]. Perceived quality is a post-purchase construct [8]. Perceived quality is essential in the measurement of consumer requirements, especially purchase intention [60]. A positive relationship between perceived quality and purchase intention towards a firm’s products and services is also identified [10]. Several studies have explored the positive impact of perceived quality on purchase intention [11]. Product perceived quality significantly impacts purchase intention. And perceived quality plays a significant role in conducting purchase intention towards international brand [63]. A cross-cultural study conducted on sports shoes in China and Korea revealed that consumers who perceive high quality will demonstrate high purchase intention [21]. Perceived quality is essential to measure consumer requirements [52] and to assess consumer behaviour, especially purchase intention [32]. A strong relationship between perceived quality and purchase intention was explored [9]. Moreover, product or service quality is an antecedent of purchase intention [11]. In the light of the above, the following hypothesis is proposed:

H5: Consumers who perceive a product as being of high quality have a higher intention to purchase.

### Price fairness and purchase intention

Price fairness is defined as what is reasonable, acceptable, and justifiable when compared with competitor prices [68]. Perceived price fairness is a major factor affecting product perception. It can lead to positive recommendations and influence purchase behaviour [13]. When assessing price fairness, a consumer may evaluate the derived value of the respective product, competitors’ prices, the price paid by other consumers, or the price-setting practices of the company [22]. The judgement of price fairness may include previous prices, rivals’ prices, and the product itself [41]. Therefore, price fairness represents the subjective evaluation of consumers. If a price seems more affordable than their internal price standards or competing prices, then customers tend to have a greater purchase intention towards the product [24]. It was also found that price fairness significantly influences purchase intention and that the former is an antecedent of the latter [35]. In the light of the above, the following hypothesis is proposed:

H6: Price fairness is positively related to purchase intention towards whitening cream.

## Methods

**Fig. 1:**
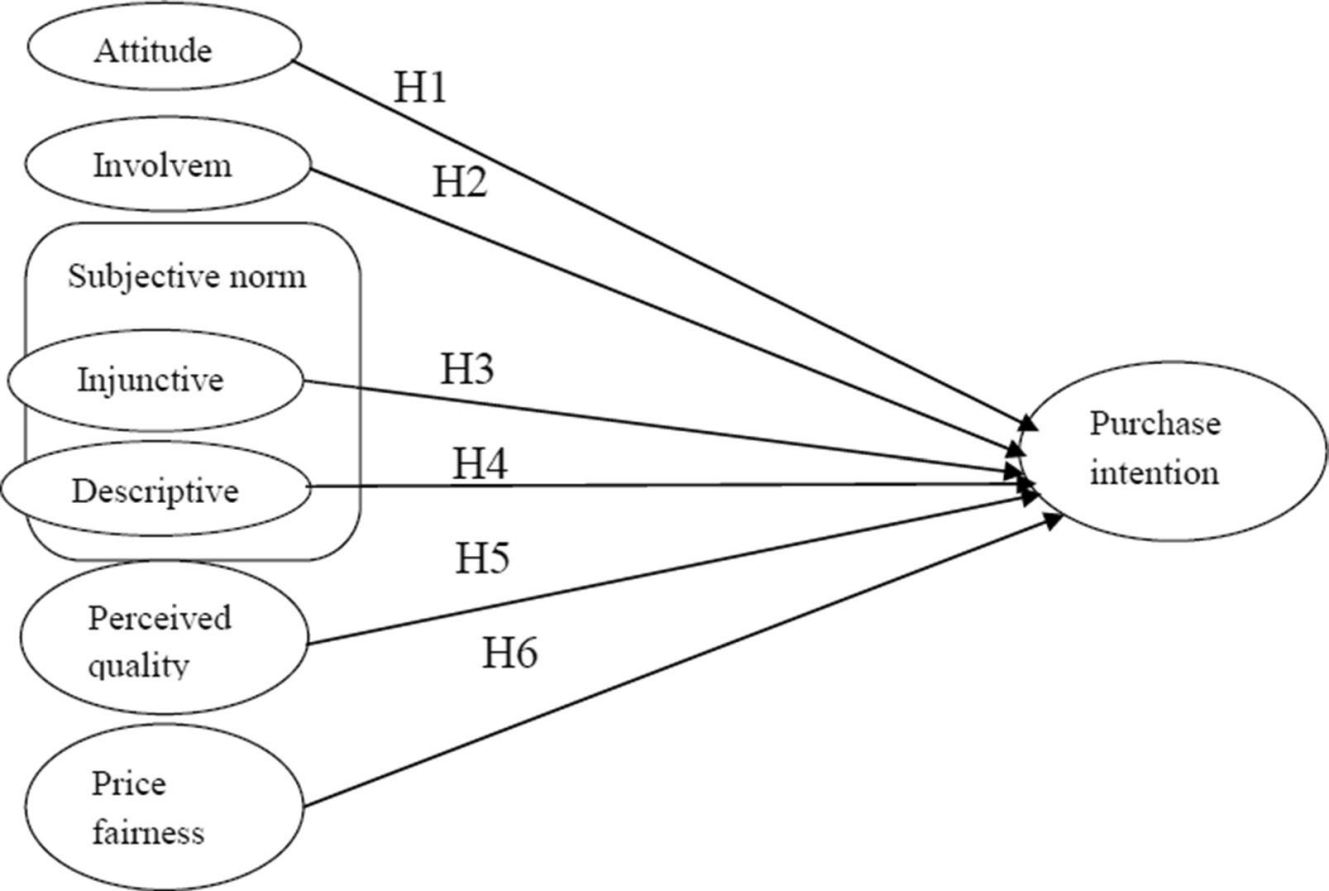
Research model

The present study’s conceptual framework (Fig. 1) consisted of five key variables: attitude, involvement, subjective norms (injunctive and descriptive), perceived quality, and perceived price fairness. Data from 275 females were collected from Sylhet City. Judgemental sampling was used because the respondents were female only. Structured questionnaire was produced using Google Forms and distributed through social media and email. A total of 300 questionnaires were sent out, and 275 usable responses were inputted. This data collection method was chosen because one-to-one interviews were not possible (owing to COVID-19). The questionnaire was piloted amongst 15 female consumers who were then using whitening cream and 5 from whitening cream businessmen and 5 from expert faculty members. The final questionnaire was prepared following feedback. A conventional translation and back-translation method was used to modify the scale in Bengali. The questionnaire comprised two sections: the first contained questions regarding the participants’ demographic profiles and the second contained questions regarding whitening cream. To assess the meaning of whitening to the participants, a question was adapted from [31]. The questionnaire was used to extract data for the independent and dependent variables (Table 1). The explanatory variable was purchase intention, which was measured using an item adapted from [43] and [49]. To measure the attitude of the participants towards purchase intention, the indicators were adapted from [51]. The participants’ interest in whitening cream was measured by items extracted from [56]. To assess the impact of injunctive norms in purchase intention, items were adapted from [20]; and to assess descriptive norms, items were adapted from [54]. To examine the impact of perceived quality on consumer purchase intention, items were extracted from [63] and [69]. Items for measuring price fairness were adapted from [39]. All the independent and dependent variables were measured on a 7-point scale ranging from 1 = *strongly disagree* to 7 = *strongly agree*. The data were analysed using SPSS and AMOS 21 versions software.

**Table 1:** Constructs and items

Construct	Item code	Scale items	Source
Attitude	ATT1	Using fairness cream is a good idea	[51]
	ATT2	Using fairness cream is wise	
	ATT3	I like the idea of using fairness cream	
	ATT4	To me, fairness creams are pleasurable	
Involvement	INV1	I find it important what kind of fairness cream I am purchasing	[56]
	INV2	It is relevant to me to purchase fairness cream	
	INV3	Purchasing fairness cream means a lot to me	
	INV4	I find it valuable to purchase fairness cream	
Injunctive norms	INJ1	Most people who are important to me think that I should use fairness cream regularly	[20]
	INJ2	Most people who are important to me expect that I should use fairness cream regularly	
	INJ3	Most people who are important to me would want me to use fairness cream regularly	
Descriptive norms	DN1	It is important to know other people's attitude regarding fairness cream	[54, 55]
	DN2	If other peoples purchase fairness cream, then it will be sensible to purchase fairness cream	
	DN3	Most people like me are using fairness cream regularly	
	DN4	Most people who are important to me use fairness cream regularly	
Perceived quality	PQ1	Fairness cream has a good functional quality	[63, 69]
	PQ2	Materials used in fairness cream are effective	
	PQ3	I am not price sensitive if the product quality is good	
	PQ4	Quality is my main concern when purchasing fairness cream	
	PQ5	Fairness cream gives me what I want	
Price fairness	PF1	I think price is important when purchasing fairness cream	[39]
	PF2	I compare prices of different brands when purchasing fairness cream	
	PF3	The price of the fairness cream is acceptable	
	PF4	The price of the fairness cream is fair	
	PF5	The price of the fairness cream is reasonable	
Purchase intention	PI1	I intend to buy fairness cream in the near future	[43, 49]
	PI2	I will try to buy fairness cream in the near future	
	PI3	I will make an effort to buy fairness cream in the near future	
	PI4	I plan to buy fairness cream regularly	
	PI5	I expect to buy fairness cream regularly	

## Results

### Respondents’ demographic characteristics

Table 2 presents information about the respondents. Most of the participants (*n* = 275) were aged between 15 and 25 (53.1%), and most (73.82%) had an income below BDT20,000. Students accounted for 62.55% of the sample. Of the 275 participants, 53.82% had attended university. The majority (74.18%) of the participants were single.

**Table 2:** Respondents’ information

Characteristics	Category	Frequency	Per cent
Age	15–25	146	53.1%
	26–35	79	28.73
	36–45	31	11.27%
	46 +	19	6.90%
Income	Below 20,000 tk	203	73.82%
	21,000 tk.-40,000 tk	56	20.36%
	41,000 tk.-60,000 tk	11	4%
	Above 60,000 tk	5	1.82%
Education	Higher secondary or less	72	26.18%
	University students	148	53.82%
	Graduate	50	18.18%
	Postgraduate	5	1.82%
Occupation	Student	172	62.55%
	Self-employed	9	3.27%
	Job holder	29	10.55%
	Housewife	65	23.64%
Marital status	Single	204	74.18%
	Married	71	25.82%
Use experience fairness cream	Yes	270	98.18%
	No	5	1.82%
Preferred product	Local	43	15.64%
	Foreign	232	84.36%
Point of purchase	Departmental store	114	41.45%
	Specialty beauty store	123	44.72%
	Online	26	9.45%
	Others	12	4.36%
Meaning of skin whitening	Changing the skin colour from black/brown to fair	123	44.72%
	Removing unwanted marks on the skin	31	11.27%
	Cleansing the skin deeply	10	3.64%
	Getting vivid and radiant skin	111	40.36%

*Source:* Primary data

Exactly 98.18% of the respondents were users of whitening cream. The other 1.82% were not currently, but they intended to; 84.36% preferred foreign whitening creams. A little under half (44.72%) of the participants preferred to purchase their creams from a specialty beauty store. They stated that they used them to change their skin colour from black/brown to fair.

### Descriptive, correlation, and discriminant validity

Prior to the further test, the descriptive statistics and correlation matrix for all the variables were evaluated. The correlation matrix shows that the variables were positively correlated and significant at the 0.01 level. The diagonal shaded and bold numbers show the square root of the average. The other values in the respective columns and rows show that all were below the square root value, thus indicating that there were no issues regarding discriminate validity (Table 3).

**Table 3:** Descriptive, correlation matrix, and square root of AVE

Construct	Mean	SD	ATT	INV	INJ	DN	PQ	PP	PI
ATT	5.7467	1.05528	.71						
INV	5.8173	.99153	.341^**^	.75					
INJ	5.6873	1.03467	428^**^	.256^**^	.79				
DN	6.0991	.90591	.182^**^	.236^**^	.202^**^	.84			
PQ	6.0564	.79775	.094	.196^**^	.152^**^	.702^**^	.85		
PP	6.0745	.86359	.289^**^	.155^**^	.228^**^	.183^**^	.107^*^	.82	
PI	6.0788	.90049	.369^**^	.371^**^	.293^**^	.303^**^	.209^**^	.148^**^	.81

**Sig. at the 0.01 and *. Sig. at the 0.05 level.

*Sources* primary data

### Reliability and validity of data

Reliability and validity analyses were conducted before the model and hypotheses tests. Cronbach’s alpha was used to assess the reliability of the measurement scales. The results were satisfactory, so the study could be run. An acceptable reliability coefficient is 0.70, but lower thresholds have been used in the literature [48]. Although the generally accepted value is at least 0.70, values lower than 0.60 are also acceptable for exploratory studies [26]. The communality value of each item was above 0.50. All the factor loading was equal to or higher than 0.64, and the average variance extracted (AVE) of each construct was higher than 0.50 (Table 4), which represents the threshold value [6].

**Table 4:** Reliability and validity of data

Constructs	Item code	Communalities	Loading	Eigen values	Cronbach's α	CR	AVE
Attitude	ATT1	0.648	0.636	7.205	0.682	0.76	0.51
	ATT2	0.696	0.726				
	ATT3	0.661	0.772				
Involvement	INV1	0.629	0.736	3.748	0.783	0.84	0.56
	INV2	0.628	0.736				
	INV3	0.681	0.751				
	INV4	0.691	0.775				
Injunctive norms	INJ1	0.756	0.805	20.454	0.763	0.83	0.62
	INJ2	0.739	0.775				
	INJ3	0.675	0.778				
Descriptive norms	DN1	0.934	0.849	1.706	0.958	0.90	0.70
	DN2	0.875	0.849				
	DN3	0.885	0.819				
	DN4	0.838	0.824				
Perceived quality	PQ1	0.908	0.905	1.491	0.934	0.91	0.72
	PQ2	0.856	0.862				
	PQ3	0.878	0.874				
	PQ4	0.703	0.743				
Price fairness	PF1	0.799	0.847	1.108	0.848	0.89	0.67
	PF2	0.757	0.862				
	PF3	0.646	0.764				
	PF4	0.698	0.793				
Purchase intention	PI1	0.690	0.792	1.019	0.809	0.85	0.65
	PI2	0.772	0.833				
	PI3	0.688	0.784				

*Source*: SPSS and AMOS output by analysing primary data

Therefore, the measurement scale items’ convergent validity was achieved. Internal consistency of the measurement scales was verified by using composite reliability, which was above 0.70, and therefore this met the threshold level suggested by researchers [26].

### Model fit

The confirmatory factor analysis (CFA) results of the estimated structural model and fit indices provided by AMOS indicated the proposed model’s adequacy to fit with the data (Table 5). Χ^2^ = 896.324 with df = 254, *p* < 0.05; RMSEA = 0.076, TLI = 0.951, CFI = 0.962, NFI = 0.935, AGFI = 0.953, GFI = 0.963. These are considered acceptable [64]. Also, the small value of RMR indicated a model fit. The columns labelled LO 90 and HI 90 contain the lower and upper limits of a 90% confidence interval for the value of RMSEA (Table 5).

**Table 5:** Model fit results

*Χ*^2^	DF	*Χ*^2^/ DF	*P* value	GFI	AGFI	NFI	CFI	TLI	RMR	RMSEA	LO 90	HI 90
896.324	254	3.529	0.000	0.962	0.953	0.935	0.962	0.951	0.065	0.076	0.066	0.082

*Source*: AMOS output by analysing primary data

### Testing the hypotheses

**Fig. 2:**
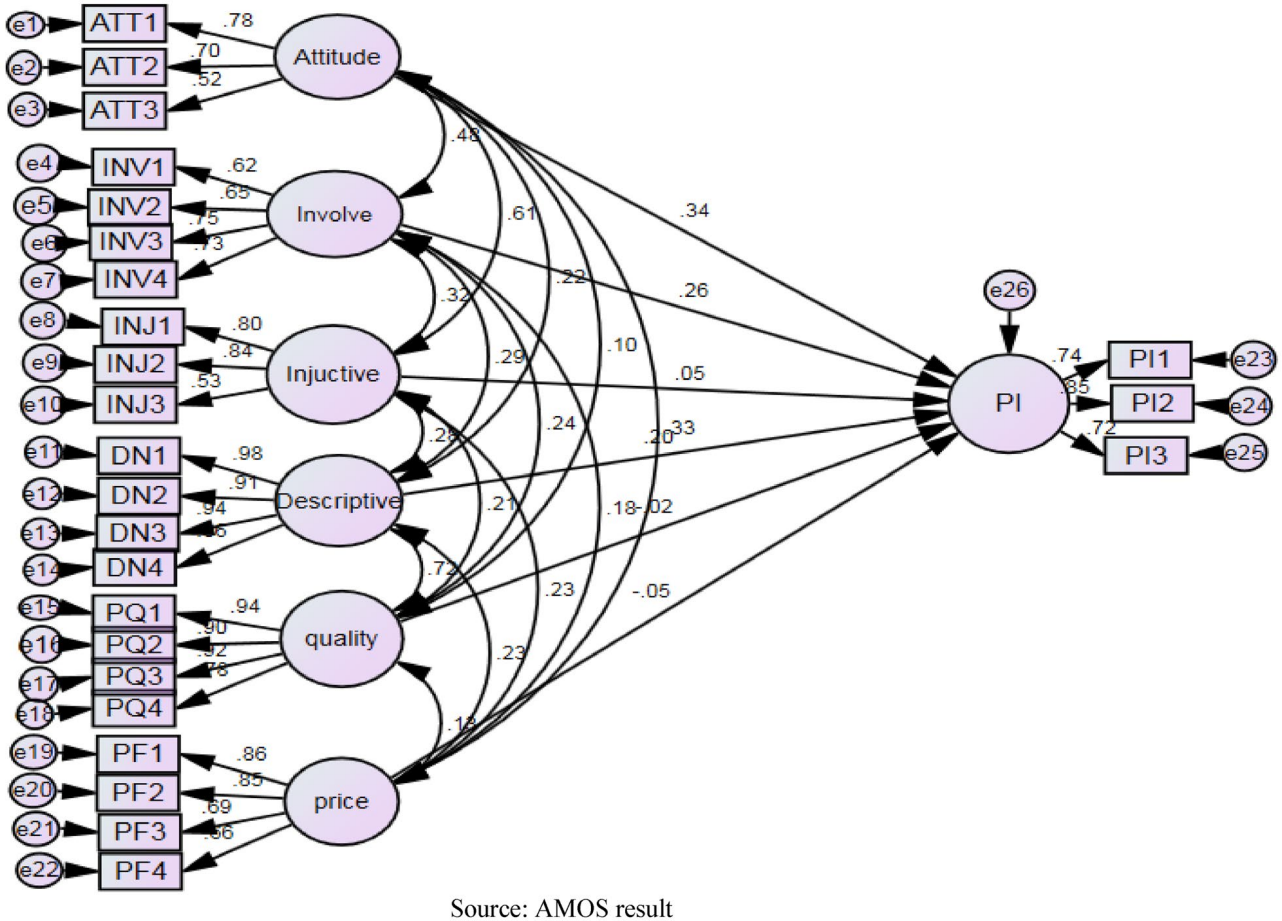
Path analysis

Based on the data analysis, standardised path coefficients (Fig. 2) and *p* values (Table 6) were provided. Hypothesis 1, which proposed that consumers with a favourable attitude towards whitening cream have a higher intention to purchase, was confirmed (path coefficient of 0.341 and *p* value = 0.004). Hypothesis 2, which proposed that consumer involvement is positively related to purchase intention towards whitening cream, was confirmed (path coefficient of 0.255 and *p* value = 0.002). Hypothesis 3, which proposed that injunctive norms are positively associated with intention to purchase whitening cream, was not confirmed (path coefficient of 0.046 and *p *value = 0.622). Hypothesis 4, which proposed that descriptive norms are positively associated with intention to purchase whitening cream, was supported (path coefficient of 0.204 and *p* value = 0.025. Hypothesis 5, which proposed that consumers who perceive a product as being of high quality have a higher intention to purchase, was not supported (path coefficient of −0.019 and *p* value = 0.831. Finally, Hypothesis 6, which proposed that price fairness is positively related to purchase intention towards whitening cream, was not confirmed (negative coefficient of −0.047 and *p* value = 0.489. In other words, the participants did not make purchase decisions based on price alone; quality may also have been considered. This finding is in keeping with [73]. It is also notable that the CR value was above 1.96, and a small standard error supported the accepted hypotheses.

**Table 6:** Hypothesis tests (path analyses)

Hypothesis	Estimate	C.R	S.E	*P* value	Result
H1: ATT→PI	0.341	2.873	0.108	0.004	Supported
H2: INV→PI	0.255	3.048	0.070	0.002	Supported
H3: INJ→PI	0.046	0.493	0.104	0.622	Not supported
H4: DN→PI	0.204	2.246	0.078	0.025	Supported
H5: PQ→PI	− 0.019	− 0.213	0.103	0.831	Not supported
H6: PF→PI	− 0.047	− 0.692	0.065	0.489	Not supported

*Source:* AMOS output by analysing primary data

### Robustness of the study

For the robustness of the study, initially a number of analyses were performed. The study looked for missing data as well as univariate and multi-variate outliers. The kurtosis and skewness of the distribution for each item were used to confirm the distribution's normality. Moreover, we used Bollen-Stine bootstrap since we have a complete data set and used here 500 samples bootstrapping and maximum likelihood estimation, and it indicated that the testing of the model is correct. A Mahalanobis distance of 1 or less indicates that the point is in the middle of the benchmark points. This study shows that all the Mahalanobis distances are less than 1. This study also used partial least square (PLS)-SEM. After evaluating the measurement model, it was confirmed that there were no concerns with reliability and validity. Then, the structural model was assessed. To perform the path analysis, 500 resamples were bootstrapped. The analysis shows that the results were similar to the AMOS SEM, where H1, H2, and H4 were accepted, and the R2 of the endogenous latent variables was 0.31. An r-square value of.12 or less indicates a small impact size, 0.13 to 0.25 indicates a medium effect size, 0.26 or more indicates a high effect size, and in this study the r-square value 0.31 suggests a high effect size [19].

## Discussion

The present study aimed to explore the role of attitude, involvement, subjective norms, perceived quality, and price fairness in influencing women’s purchase intention towards skin-whitening cream. An extensive review of the literature resulted in six hypotheses, from which the study’s structural model was derived. This was tested by examining data collected from 275 females.

A strong positive relationship was found between attitude and purchase intention. A positive attitude was a crucial determinant. A more favourable attitude directed the participants towards positive purchase intention. The participants considered purchasing whitening cream as a good idea and thought that using it was a sensible idea. The findings relating to attitude and purchase intention were in line with previous studies[62]; [33].

The results suggested that consumer involvement had a significant relationship with purchase intention. According to the literature, consumer involvement is a strong determinant of purchase intention [14]. The participants were more involved with the type of whitening cream they were purchasing. They regarded their purchase as valuable and personally significant.

We assessed subjective norms in two parts (injunctive and descriptive). Injunctive norms were insignificant in purchase intention. This implied that the participants’ purchase intentions were not influenced by the opinions of others—an insignificant relationship that confirmed previous studies [36]. A significant relationship between descriptive norms and purchase intention was revealed. This finding is supported by two other researchers [27]. It shows that the items studied under descriptive norms were strong enough to predict the participants’ purchase intention. It can therefore be stated that the participants thought neither about others’ approval nor disapproval of their purchasing behaviours. Following descriptive norms, people are normally interested in knowing what others do and seek to behave accordingly.

An insignificant relationship was discovered between perceived quality and purchase intention. This finding is in keeping with other research also [46]. It may have depended on situational factors because, although the supplied product or service may be of the highest standard, the participants may have had an expectation that could not be met under any circumstances.

## Implications

The present study contributes to the current literature by exploring the relationship between attitudes, involvement, subjective norms, perceived quality, perceived price, and purchase intention. The positive relationship between attitude and purchase intention suggests that the participants who regarded whitening cream as practically advantageous and pleasurable had a strong intent to purchase. Therefore, to attract consumers, it is imperative to build formative attitude clues. The findings provide strong theoretical support regarding attitude formation and how it influences consumer behaviour. Attitude has significant implications for whitening cream marketing; advertisers should try to influence attitude formation clues through storytelling. But this endeavour should be undertaken ethically; marketers should not deceive consumers by suggesting that whitening cream can make them beautiful (which may have some racist undertones) or confer other advantages.

The significant relationship between descriptive norms and purchase intention suggests that the participants were interested in what other people do. To encourage purchase intention, marketers could use brand endorsements to enhance credibility. The concept of descriptive norms could be exploited to this end. Moreover, the character of the endorser is crucial here.

The significant relationship between involvement and purchase intention revealed that the participants considered whitening cream to be valuable and important. Marketers should therefore attempt to persuade non-involved consumers to purchase by using tools such as eye-catching audio-visuals on TV advertisements, interactive billboards, scratch options on packaging for gift giveaways, and so on.

The present study clarifies issues concerning whitening products for consumers and may help marketers to track current market trends and purchase intention. It provides demographic information that will be valuable in the targeting of customers.

## Limitations and future research

The present study has several limitations. The data only covered females from Sylhet City. Because purchase intention and preferences may vary according to gender and region, future researchers could study broader populations in both respects. Also, the data were collected using non-probability convenience sampling, which means that the results cannot be generalised for that reason alone. Future studies could use probability sampling (e.g. random sampling) and carry out longitudinal studies to address this shortcoming. It would also be advisable to examine the impact of different moderating and mediating effects on a wider range of variables. The findings cannot be applied to other sectors because each industry is distinct, but the model used for the present study could be used to analyse other beauty services.

In summary, while the present study contributes to the literature on the skincare industry by providing a deeper understanding of a particular aspect of it, future researchers should address some of its shortcomings.

## Conclusions

Marketers have cultivated in both men and women a latent desire to have lighter skin because of the extraordinary advantages this is considered to confer. In future, the focus should be on nourishing, cleansing, and rejuvenating skin rather than making it white. Also, the government should take steps to regulate the production of harmful creams. High prices could deter consumers from purchasing products that are a health hazard. There are some signs that marketers are changing their approach. For instance, Dove promotes the idea of *real beauty*, an admirable initiative that may help to shift the paradigm [53].

The present study offers insights into customer purchase intention towards whitening cream by investigating the relationship between multiple variables (i.e. attitude, involvement, injunctive and subjective norms, perceived quality, and price fairness) and participants’ purchase intention. The results reveal that perceived quality, price fairness, and injunctive norms had an insignificant relationship with purchase intention. By contrast, attitude, descriptive norms, and involvement had a significant relationship with purchase intention. The present study provides the basis on which to build a customer-centric approach towards the question of purchase intention. The information it provides will help both marketers and scholars in their work. Marketers can explore the factors that are crucial in encourage consumers to buy whitening products—intention in particular. Accurate perception of consumer purchase intention can give firms the power to set premium prices and secure consumer loyalty. Meanwhile, scholars can use the study as a foundation on which to investigate different populations, related ethical issues, and so on.

## Abbreviations

SEM: Structural equation modelling
AMOS: Analytical moment of structures
SPSS: Statistical Package for the Social Sciences
AVE: Average variance extracted
CFA: Confirmatory factor analysis
DF: Degrees of freedom
RMSEA: Root-mean-square error of approximation
TLI: Tucker–Lewis index
RMR: Root-mean-square residual
GFI: Goodness-of-fit index
AGFI: Adjusted goodness-of-fit index
CFI: Comparative fit index
NFI: Normed fit index
CR: Composite reliability
SE: Standard error
PLS: Partial least square
